# Initial Stage Carbonization of γ-Fe(100) Surface in C_2_H_2_ under High Temperature: A Molecular Dynamic Simulation

**DOI:** 10.3390/ma14205957

**Published:** 2021-10-11

**Authors:** Yu Sun, Ling Wang, Hao Wang, Ziqiang He, Laihao Yang, Xuefeng Chen

**Affiliations:** 1 School of Mechanical Engineering, Xi’an Jiaotong University, Xi’an 710049, China; yu.sun@xjtu.edu.cn (Y.S.); wl18437670360@stu.xjtu.edu.cn (L.W.); wh0419@stu.xjtu.edu.cn (H.W.); chenxf@xjtu.edu.cn (X.C.); 2 State Key Laboratory for Manufacturing Systems Engineering, Xi’an Jiaotong University, Xi’an 710049, China; 3 AECC Beijing Institute of Aeronautical Materials, Beijing 100095, China; hezq2005@163.com

**Keywords:** austenite, carbonization, molecular dynamics, microstructure

## Abstract

In the present work, initial stage carbonization of γ-Fe(100) surface in C_2_H_2_ from 1000 K to 1600 K has been investigated by a molecular dynamic (MD) simulation, based on which the atomic mechanism of initial stage carbonization was provided. The absorption of C and H atoms during the carbonization process under different temperatures was analyzed. The related distributions of C and H atoms in carbonized layer were provided. The results manifested that higher temperature enhanced the inward diffusion of C and H, meanwhile caused the desorption of H atom. Furthermore, the effect of preset polycrystal γ-Fe on the carbonization process has been discussed, indicating a promoting role to the absorption and inner diffusion of C and H atom. The results of this study may support the optimal design of high-performance steel to some extent.

## 1. Introduction

Iron-based alloys are generally accompanied with carbides, which are of great interest since the extensive application of steel in industry [1]. With long time development, carbonization is still an indispensable way for the surface treatment and strengthening of iron so far. The related atomistic scale mechanism and kinetics of carbonization play an influential role on the property of steel, while these are difficult to be detected by experimental facilities such as X-ray diffraction, scanning electron microscopy and transmission electron microscopy [2,3]. In addition, due to the increasing use of nanoscale materials and components, the mechanism of initial stage reaction occurring within atomistic scale determines their future performance [4,5,6]. In the field of nanoscale behaviors analysis, molecular dynamics (MD) simulation shows its superiority and well application.

Regarding to the previous work on Fe-C alloy by MD simulations, most of them focused on the carbon diffusion in bcc or fcc iron [7,8,9,10,11]. The mutual effects between carbon interstitial and defects in iron have been intensely studied [12,13,14,15,16,17,18,19]. Furthermore, some of them were well devoted to the phase transformation mechanisms in Fe-C alloys [20,21,22,23,24]. The above-mentioned investigations revealed the atomistic mechanisms on the carbon interstitial in Fe. To improve the carbonization efficiency and related performance of Fe-C, the higher C concentration supplement is necessary. Since the C_2_H_2_ has the highest C concentration providing a noteworthy ability when carbonized with γ-Fe, it is one of the promising carbonization gases. During the carbonization process of γ-Fe in C_2_H_2_, the absorption of C and H atoms could induce lattice mismatch and chemical reaction with Fe. Hence, it is significant to study the atomistic system of Fe-C-H.

Recently, the bond order potential (BOP) for Fe-C-H has been proposed by Zhou et al. [25], which making MD simulation on carbonization of γ-Fe in C_2_H_2_ become possible. By this BOP framework, Jiao et al. analyzed the effects of hydrogen on the deformation behavior of fcc Fe-C crystal [26]. Deep understanding on the deformation mechanism of fcc Fe-C single crystal with nanovoid was given. However, the absorption of C and H atoms into Fe lattice under high temperature has not been considered. In the present work, by BOP based MD simulation, the absorption and related distribution of C and H atoms in fcc Fe were researched. The effect of pre-existed polycrystal on the carbonization process has been discussed. 

The paper is organized as follows: Section 2 gives the computational details including model setup and simulation parameters; Section 3 provided the results discussion including initial stage of carbonization process, structure of carbonized layer and effect of preset polycrystal on initial carbonization.

## 2. Computational Details

**Figure 1:**
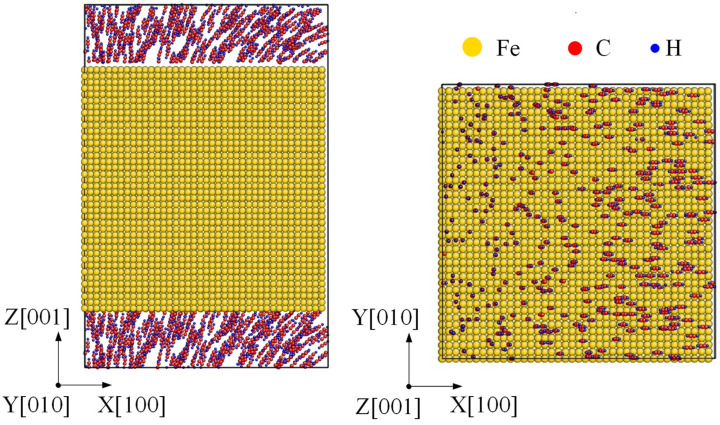
Initial carbonization model. A γ-Fe(100) nanofilm filling with C_2_H_2_ molecules at top and bottom surfaces.

The initial carbonization model was set up as follows. Firstly, an fcc Fe with size as 73 × 73 × 73 Å along [100], [010] and [001] was built, which contains 32,000 atoms. To obtain Fe(100) nanofilm with top and bottom as surface, the simulation box was enlarged to 108.3 Å along *z* direction. Then, C_2_H_2_ molecules were distributed randomly near the top and bottom surfaces. Since the time scale of MD simulation was too short to the nanosecond, a relatively large acetylene gas pressure was set as 200 atm to decide the number of C_2_H_2_ molecules. Hence the chemical reaction could be completed within MD simulation time [27,28,29]. The initial model of γ-Fe(100) nanofilm surrounding with C_2_H_2_ is shown in Figure 1.

To probe the reactive process of Fe in C_2_H_2_, the latest developed bond-order potential (BOP) was applied to describe the interaction of Fe-C-H. The Fe-C-H potential was combined by Fe-C, Fe-H and C-H bond order potentials. We used the BOP I, which has been proved to provide the exact lattice constant, cohesive energy, elastic constants, surface energy of α-Fe and γ-Fe comparing with experimental results [30,31] and density functional theory calculations [32,33,34]. Significantly, the diffusion kinetics of C and H were correctly described by such a potential. By using BOP I, the carbonization of Fe by C_2_H_2_ was simulated in the present work via an open-source LAMMPS (Version: https://www.lammps.org/ accessed on 5 May 2020) code [35].

To maintain fcc structure, the temperature was set from 1000 K to 1600 K. A time step of 0.5 fs was used, and the total simulation time was 3 ns long enough to provide an initial reaction process. A temperature damping constant of 50 fs for NVT (canonical) ensemble under Nose-Hoover heat bath was applied for all the cases. The distributions of C/H atoms were obtained by meshing Fe along *z* direction from the surface with thickness as a monolayer [36,37].

## 3. Results and Discussion

### 3.1. Initial Stage of Carbonization Process

**Figure 2:**
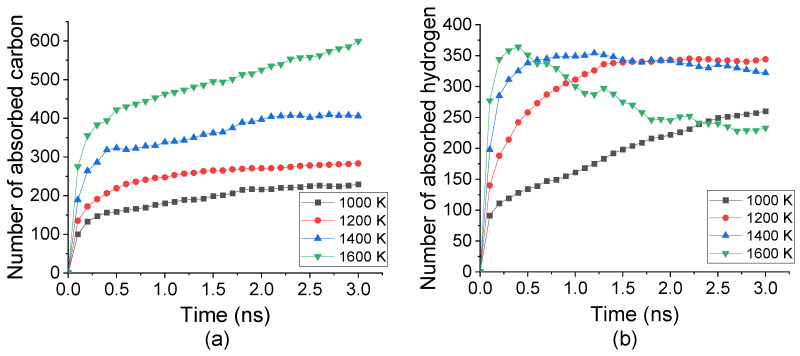
Numbers of (**a**) C atoms and (**b**) H atoms absorbed into top carbonized layer during carbonization process under temperature from 1000 K to 1600 K.

Under temperature from 1000 K to 1600 K, C_2_H_2_ molecules were decomposed into C and H atoms then absorbed into Fe crystal. Accompanying with the carbonization process, the number of absorbed C and H atoms into the top surface under different temperatures was tracked, as shown in Figure 2. The absorption rate of both C and H atoms remarkably increased from the beginning to nearly 0.2 ns, indicated by the curve gradient. Such a rate was enhanced by increasing the temperature from 1000 K to 1600 K. However, the temperature displaced an inverse effect for C and H atoms after 0.2 ns. The number of absorbed C still grew up slowly after 0.2 ns. It was raised by increasing the temperature, which was nearly three times under 1600 K comparing with that under 1000 K. The number of absorbed H atoms was increased from 100 to 250 under 1000 K whereas decreased from 320 to 260 under 1600 K. This indicated that the absorption of C atoms was promoted by temperature increasing while H atoms may desorb from Fe under a higher temperature. 

**Figure 3:**
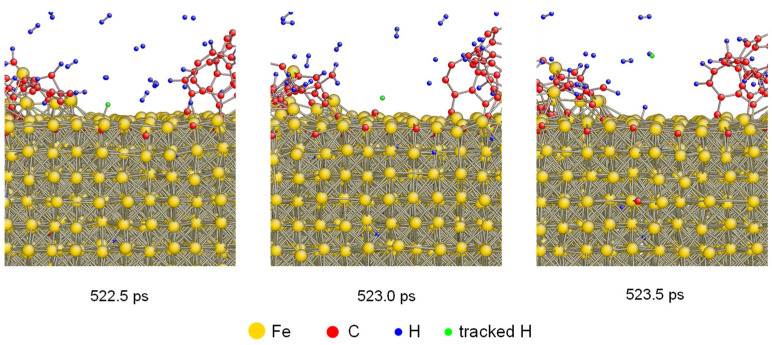
Atomistic configurations of H desorption from the carbonized layer under 1600 K. One H atom was tracked by coloring as green.

To investigate the H desorption under 1600 K in detail, the tacking of H atoms was performed. The atomistic configurations of one H atom desorbing from carbonized layer is shown in Figure 3. There was an absorbed H atom colored as green which was bonded with Fe at 522.5 ps. After that, it was desorbed from the carbonized Fe and bonded with another H atom generating a hydrogen molecule. The desorption of H atoms was also demonstrated by previous results based on both simulation and experiment [38,39,40].

**Figure 4:**
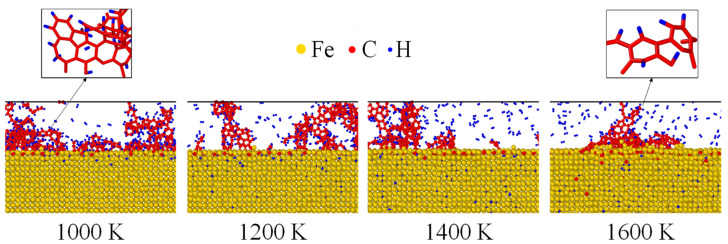
Atomistic configurations of Fe nanofilm after carbonized for 3 ns. The residual C atoms formed benzene rings, showing as the zoomed view at the top.

The atomistic configurations of Fe after carbonized for 3 ns was shown in Figure 4. Fe, C and H atoms were, respectively, colored by yellow, red and blue. Under 1000 K, almost all the C and H atoms were located near the surface while they were transported to inner layers as the temperature increases. As indicated by Figure 4, thermal activation enhanced the inward diffusion of both C and H atoms. Furthermore, the residual C atoms gathered and reacted to form benzene rings under all temperatures, shown as the zoomed view of Figure 4. Such a chemical phenomenon has also been observed experimentally [41,42].

### 3.2. Structure of Carbonized Layer

**Figure 5:**
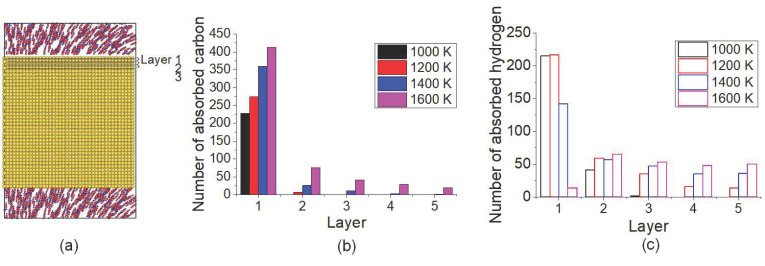
(**a**) The schematic of the monolayer of Fe crystal. Distributions of (**b**) C atoms and (**c**) H atoms in the top surface layer after carbonization for 3 ns under different temperatures.

To clarify the temperature effect on C/H distribution in carbonized layer, the number of C and H atoms located in monolayer 1 (the first from top surface) to 5 (the fifth from top surface) after carbonized for 3 ns under different temperatures was calculated, shown as in Figure 5. The schematic of monolayer of Fe crystal is presented in Figure 5a. As in Figure 5b, it is shown that most C atoms existed at the topmost layer under all temperatures. Under 1600 K, the number of C atoms in all the monolayers was the largest one. From Figure 5c, it seems that H atoms are much more transported to the inner layers, especially under 1600 K. The distribution of C and H atoms implied that the inward transport of both C and H atoms was intensified by temperature increasing, more notable for H atoms. Even though the desorption of H under 1600 K caused the decreasing of the total number of the absorbed H atoms in the carbonized layer, more H atoms stayed at inner layers compared with lower temperature. 

**Figure 6:**
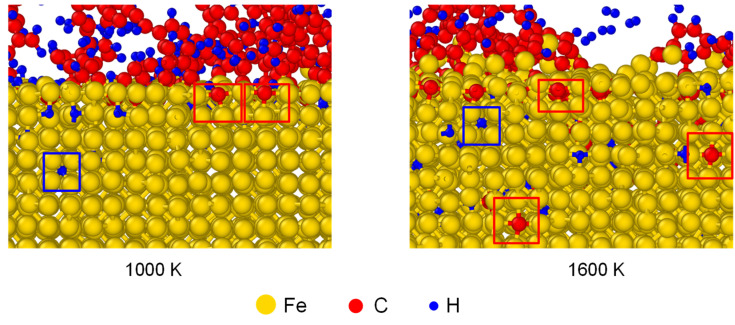
The interstitial sites of C and H atoms in Fe after carbonized for 3 ns under 1000 K and 1600 K. The structures of austenite formed in γ-Fe are put in red frames.

**Figure 7:**
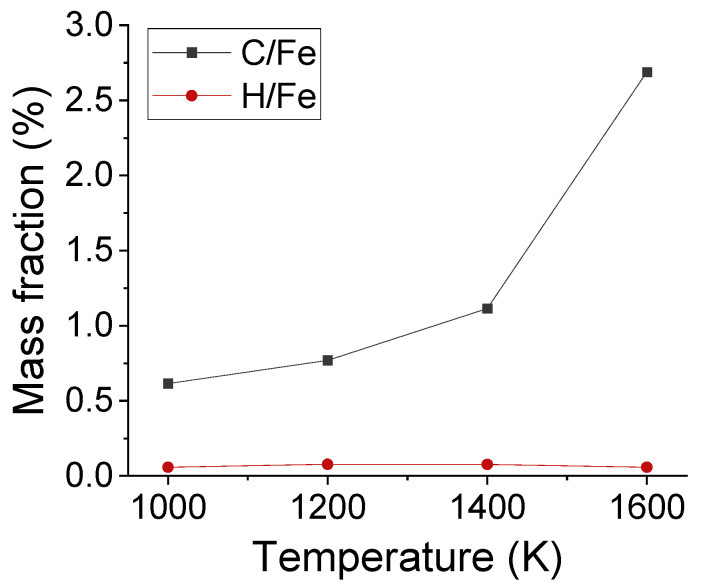
The average mass fraction of C/Fe and H/Fe ratios for the first five Fe layers after carbonization for 3 ns under different temperatures.

Apart from the distribution of interstitial atoms, the interstitial sites of C and H atoms were observed in Fe after carbonized for 3 ns, as shown in Figure 6. As framed out by the red boxes, the absorbed C occupied the interstitial sites of austenite structure. C atoms stayed at the surface under 1000 K, but migrated to deeper layers under 1600 K. To compare the solubility of C and H atoms in austenite structure, the average mass fraction of C/Fe and H/Fe ratios for the first five Fe layers has been calculated, as shown in Figure 7. The mass fraction of C increased with temperature and was in the range of 0.7% to 1.5% from 1000 K to 1420 K. It agrees with experimental result which is 0.77% under 727 ºC and 2.11% under 1148 ºC.

### 3.3. Effect of Preset Polycrystal on Initial Carbonization

**Figure 8:**
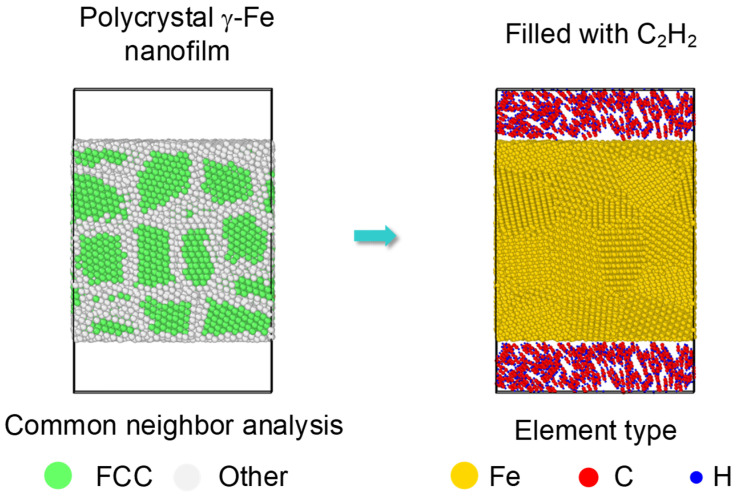
Initial carbonization model of a preset polycrystal. A polycrystal with a grain size as 2.3 nm is set up first, shown by common neighbor analysis on the left. Then the top and bottom surfaces are filled with C_2_H_2_ molecules, shown by element type on the right.

Moreover, almost all the samples used in industry experienced a combined reinforcement process, such as laser shocking followed by carbonization. After a laser shocking process, the surface of Fe turns to polycrystal type, leading to an effect of preset polycrystal on carbonized layer. Relatively, an initial model of a preset polycrystal Fe was built as Figure 8. Three crystal grains divided by two grain boundaries was formed along each axis with a grain size of nearly 2.3 nm. Then C_2_H_2_ molecules were randomly filled into the top and bottom space near the surface. Corresponding simulations were carried on for the polycrystal Fe under 1000 K to 1600 K by the same setting as monocrystal type.

**Figure 9:**
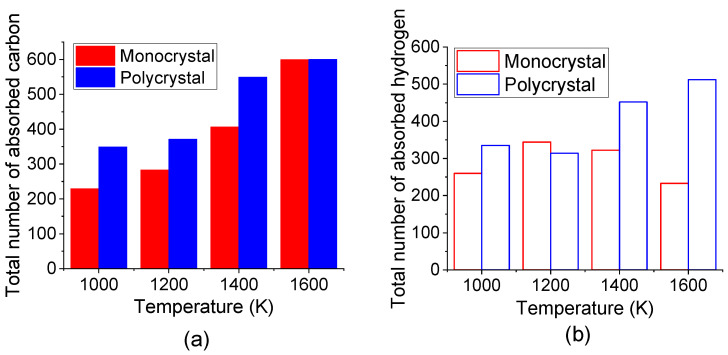
Total number of absorbed (**a**) C atoms and (**b**) H atoms in top carbonized layer of monocrystal and polycrystal Fe after carbonized for 3 ns under different temperatures.

The comparison of the total number of absorbed C and H atoms in the top carbonized layer of monocrystal and polycrystal Fe after carbonized for 3 ns under different temperatures was shown in Figure 9. It is suggested that more C and H atoms are absorbed into polycrystal Fe under all temperatures. A previous study by reactive molecular dynamics has stated that the pre-existing defects promoted carbon diffusion [43]. Since the atom in the grain boundary has a lower density than that inside the grain, grain boundary could supply more space for interstitial atoms to move. Here, in this study, the results also displayed that the grain boundary may act as an energy-favorable configuration for the absorption of C and H atoms.

**Figure 10:**
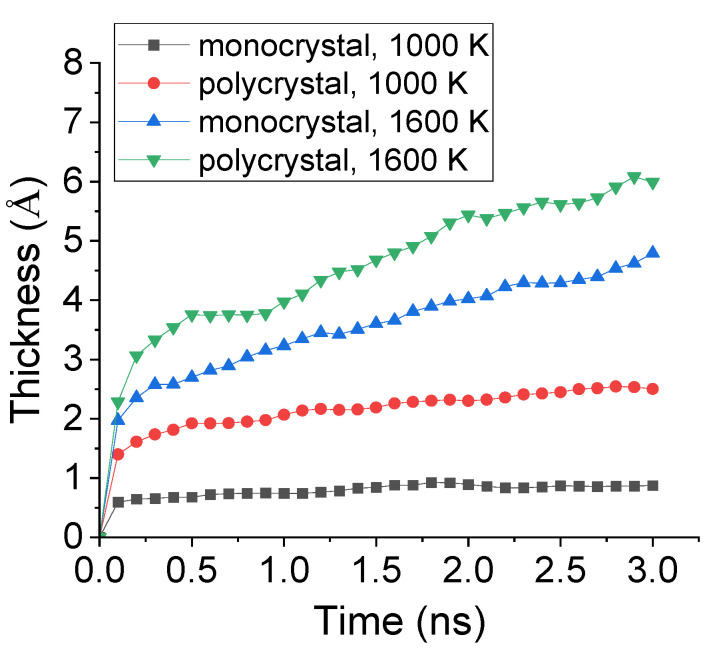
Evolution of carbonized thickness during carbonization process of monocrystal and polycrystal Fe under 1000 K and 1600 K.

To further study the effect of preset polycrystal on the carbonized layer, the time evolution of carbonized thickness during carbonization process was calculated. The thickness was decided by the center of mass concerning all the absorbed C atoms. The comparison of the carbonized thickness of monocrystal and polycrystal Fe under 1000 K and 1600 K was shown in Figure 10. It is observed that, under 1000 K, the carbonized thickness for both monocrystal and polycrystal types greatly increased before 0.2 ns and, afterwards, remained a relatively low level. In addition, the thickness of the carbonized layer of polycrystal Fe was nearly 4 times to that of monocrystal type. However, under 1600 K, although the total number of absorbed C atoms was similar to what shown in Figure 9, the carbonized thickness increased because the inner transportation was enhanced by preset polycrystal.

## 4. Conclusions

In this paper, by means of MD simulation with BOP, the initial stage carbonization of γ-Fe(100) surface in C_2_H_2_ under high temperature has been studied. It is shown that a higher temperature enhanced the absorption of C atoms but led to the desorption of H atoms from Fe crystal. C atoms occupied the interstitial sites of austenite structure, and their inner transportation were promoted by increasing the temperature. Furthermore, the effect of polycrystal on carbonization has been investigated, playing as a facilitation to reinforce the absorption and inward diffusion of C atoms. We believe the present results could theoretically promote the understanding and improvement on high-temperature carbonization of steel.
